# The Pro12Ala Polymorphism of the Peroxisome Proliferator-Activated Receptor Gamma Gene Modifies the Association of Physical Activity and Body Mass Changes in Polish Women

**DOI:** 10.1155/2014/373782

**Published:** 2014-10-13

**Authors:** Aleksandra Zarebska, Zbigniew Jastrzebski, Pawel Cieszczyk, Agata Leonska-Duniec, Katarzyna Kotarska, Mariusz Kaczmarczyk, Marek Sawczuk, Agnieszka Maciejewska-Karlowska

**Affiliations:** ^1^Faculty of Tourism and Recreation, Gdansk University of Physical Education and Sport, Kazimierza Gorskiego Street 1, 80-336 Gdansk, Poland; ^2^Faculty of Physical Education and Health Promotion, University of Szczecin, Piastow 40B, 71-065 Szczecin, Poland

## Abstract

Peroxisome proliferator-activated receptor γ is a key regulator of adipogenesis, responsible for fatty acid storage and maintaining energy balance in the human body. Studies on the functional importance of the *PPARG* Pro12Ala polymorphic variants indicated that the observed alleles may influence body mass measurements; however, obtained results were inconsistent. We have decided to check if body mass changes observed in physically active participants will be modulated by the *PPARG* Pro12Ala genotype. The genotype distribution of the *PPARG* Pro12Ala allele was examined in a group of 201 Polish women measured for selected body mass variables before and after the completion of a 12-week training program. The results of our experiment suggest that *PPARG* genotype can modulate training-induced body mass measurements changes: after completion of the training program, Pro12/Pro12 homozygotes were characterised by a greater decrease of body fat mass measurements in comparison with 12Ala allele carriers. These results indicate that the *PPARG* 12Ala variant may impair the training-induced positive effects on body mass measurements; however, the detailed mechanism of such interaction remained unclear and observed correlation between *PPARG* genotype and body mass differential effects should be interpreted with caution.

## 1. Introduction

Peroxisome proliferator-activated receptor *γ* (PPAR*γ*) is a transcriptional factor involved mainly in controlling the carbohydrate and lipid homeostasis. This protein is considered as a key regulator of adipogenesis, responsible for fatty acid storage and maintaining energy balance in the human body through the control of complex metabolic networks of insulin-dependent pathways. The discovery that the lipid-activated PPAR*γ* nuclear receptor is essential for adipocyte development has contributed to our understanding of adipose tissue biology and the role of PPAR*γ* regulation in obesity-related diseases such as hyperlipidemia, insulin resistance, type 2 diabetes mellitus, obesity, and cardiovascular diseases [1].

In humans, PPAR*γ* is encoded by the* PPARG* gene located on chromosome 3. As a consequence of different promoters usage (including the internal promoter) as well as alternative splicing, at least four mRNA transcripts that differ at their 5′ ends are produced [2–4]. The PPAR*γ* isoforms formed on their basis—*γ*1, *γ*3, and *γ*4—are identical because they are based on the information contained in exons 1 to 6, which are a common element for all produced transcripts and only the *γ*2 form has 28 additional N-terminal amino acids, encoded by exon B of the* PPARG* gene, which is located exactly at the differentiative 5′ end of mRNA molecule [5]. Within the B exon of human* PPARG* gene, the polymorphic point C/G (rs1801282) is found. This nucleotide substitution was first identified by Yen et al. [6] as being responsible for the substitution of proline in position 12 of the PPAR*γ*2 protein for alanine (Pro12Ala substitution). The location of this amino acid substitution point in the PPAR*γ*2 AF-1 domain, controlling the ligand-independent ability to activate target gene expression, allowed us to hope that the observed* PPARG* allelic forms (usually described as allele Pro12 identical with the C allele and the 12Ala allele corresponding to the G allele) will be of functional importance. This, in turn, might influence human physiology, as conformed in many experiments.* In vitro* studies revealed that the presence of the* PPARG* 12Ala allele is related to a diminished affinity of PPAR*γ*2 for the PPRE (peroxisome proliferator response element) sequence in target gene promoters [7], which results in a decrease of their expression level, which was also corroborated by* in vivo* studies [8–10]. Moreover, it was revealed that the 12Ala allele has an insulin-sensitising effect on the liver and skeletal muscles [7, 11]. Such increased sensitivity to insulin, influenced by the presence of the* PPARG* 12Ala allele, caused suppression of lipolysis in adipocytes and diminished the release of free fatty acids [12]. The restricted availability of this molecular fuel causes a specific shift of energy metabolism to anaerobic metabolism with a simultaneous increase of glucose consumption in active skeletal muscles, as has been proved in studies showing an increase of glucose consumption in skeletal muscles after insulin stimulation in carriers of the 12Ala allele and a considerable decrease of flexibility in energy substrate selection in Pro12/Pro12 homozygotes [13, 14].

Our scientific team have previously demonstrated that the* PPARA* intron 7 and* PPARGC1A* Gly482Ser polymorphisms are associated with endurance athlete status in Polish athletes [15–18]. Considering the results of studies on the functional importance of the* PPARG* Pro12 and 12Ala alleles, we assumed that the presence of the 12Ala allele in a genome, as an element of a system favouring glucose consumption mainly in anaerobic metabolism, ought to be especially beneficial for athletes subjected to short-term physical exertion of maximal intensity, during which muscle energy is produced mainly in anaerobic metabolism. We conducted a cross-sectional study including genotyping of the* PPARG* Pro12Ala SNP in a very large and heterogeneous cohort of athletes [19] and proved that the* PPARG* 12Ala allele frequency is much higher in Polish athletes recruited from sprinters (100–400 m distance), representatives of typical power sports (weightlifting and power lifting), throwers (shot put, discus throw, javelin throw, and hammer throw) and jumpers (long jump, triple jump, high jump, and pole vault). Our results corresponded with the results of genotyping performed in a similar cohort of Russian athletes, in which an increased 12Ala allele frequency in sprinters, throwers, and weightlifters was observed [20]. Moreover, these analyses proved the presence of the 12Ala allele fosters muscle fiber hypertrophy, which suggests that this* PPARG* variant contributes to the occurrence and development of such motor skills as strength and speed [20].

Considering the aforementioned facts and promising results of our previous analyses, we have decided to continue with the study on possible* PPARG* Pro12Ala variants and human physical traits associations. Taking into account the PPAR*γ* role in adipocyte development, we have decided to check if body mass changes observed in physically active participants will be modulated by the* PPARG* Pro12Ala genotype. To test this hypothesis, we have performed a genetic association study that aimed to detect a correlation between the Pro12Ala genetic polymorphism and selected body composition measurements. Therefore, we have examined the genotype distribution of the* PPARG* Pro12Ala allele in a group of Polish women measured for selected body mass and body composition variables before and after the completion of a 12-week training program. Our statistical analyses revealed that there were four significant* PPARG* genotype × training interactions (for tissue impedance, fat mass percentage, fat mass, and FFM), indicating the potential role of* PPARG* Pro12/Pro12 genotype in training-induced body mass measurements changes.

## 2. Materials and Methods

### 2.1. Ethics Statement

The procedures followed in the study were conducted ethically according to the principles of the World Medical Association Declaration of Helsinki and ethical standards in sport and exercise science research. The procedures followed in the study were approved by the Ethics Committee of the Regional Medical Chamber in Szczecin (Approval number 09/KB/IV/2011). All participants were given a consent form and a written information sheet concerning the study, providing all pertinent information (purpose, procedures, risks, and benefits of participation). The potential participant had time to read the information sheet and the consent form. After ensuring that the participant had understood the information, every participant gave written informed consent (signed consent form) to genotyping on the understanding that it was anonymous and that the obtained results would be confidential. The experimental procedures were conducted in accordance with the set of guiding principles for reporting the results of genetic association studies defined by the Strengthening the Reporting of Genetic Association studies (STREGA) Statement [21].

### 2.2. Participants

201 Polish Caucasian women aged 21 ± 1 years (range 19–24) met the inclusion criteria and were included in the study. None of these individuals had engaged in regular physical activity in the previous 6 months. They had no history of any metabolic or cardiovascular diseases. Participants were nonsmokers and refrained from taking any medications or supplements known to affect metabolism. Prior to the start of the training phase participants were asked to keep a balanced diet of approximately 2000 kilocalories a day.

### 2.3. Body Composition Measurements

All participants were measured for selected body mass and body composition variables before and after the completion of a 12-week training period. Body mass and body composition were assessed by bioimpedance method (body's inherent resistance to an electrical current) with the use of the electronic scale “Tanita TBF 300M” (Horton Health Initiatives, USA). The device was plugged in and calibrated with the consideration of the weight of the clothes (0.2 kg). Afterwards, data regarding age, body height, and sex of the subject was inserted. Then, the subjects stood on the scale with their bare feet on the marked places without leaning any body part. The device analyses body composition based on the differences of the ability to conduct electrical current by body tissues (different resistance) due to different water content. Body mass and body composition measurements taken with the use of the electronic scale “Tanita” are as follows: total body mass (kg), fat free mass (FFM, kg), fat mass (kg), fat mass percentage (% FM), body mass index (BMI = body mass (kg) × body height (m^2^)^−1^), tissue impedance (Ohm), total body water (TBW, kg), and basal metabolic rate (BMR, kJ or kcal).

### 2.4. Training Phase

The training stage was preceded by a week-long familiarization stage, when the examined women exercised 3 times a week for 30 minutes, at an intensity of about 50% of their HRmax. After the week-long familiarization stage, the proper training has started. Each training unit consisted of a warm-up routine (10 minutes), the main aerobic routine (43 minutes), and stretching and breathing exercise (7 minutes). The main aerobic routine was a combination of two alternating styles—low and high impact. Low impact style comprises movements with at least one foot on the floor at all times, whereas high impact styles include running, hopping, and jumping with a variety of flight phases [22]. Music of variable rhythm intensity (tempo) was incorporated into both styles. A 12-week program of low-high impact aerobics was divided as follows: (i) 3 weeks (9 training units), 60 minutes each, at about 50–60% of HRmax, tempo 135–140 BPM, (ii) 3 weeks (9 training units), 60 minutes each, at 50–60% of HRmax, tempo 135–140 BPM, (iii) 3 weeks (9 training units), 60 minutes with the intensity of 60%−70% of HRmax, tempo 140–152 BPM, and (iv) 3 weeks (9 training units), 60 minutes with an intensity of 65%−75% of HRmax, tempo 140–152 BPM. All 36 training units were administered and supervised by the same instructor.

### 2.5. Genetic Analyses

The buccal cells donated by the subjects were collected in Resuspension Solution (GenElute Mammalian Genomic DNA Miniprep Kit, Sigma, Germany) with the use of sterile foam-tipped applicators (Puritan, USA). DNA was extracted from the buccal cells using a GenElute Mammalian Genomic DNA Miniprep Kit (Sigma, Germany) according to the manufacturer's protocol. All samples were genotyped in duplicate using an allelic discrimination assay on a StepOne Real-Time Polymerase Chain Reaction (RT-PCR) instrument (Applied Biosystems, USA) as previously described [19].

### 2.6. Statistical Analyses

Allele frequencies were determined by gene counting. A *χ* ^2^ test was used to test the Hardy-Weinberg equilibrium. To test the influence of* PPARG* Pro12Ala polymorphism on training response, the 2 × 2 mixed-design ANOVA test was used. Additionally, normality Kolmogorov-Smirnov test was used to check for data normality, and post hoc Tukey test was applied when interaction was significant and was used to perform pair-wise comparisons. The level of statistical significance was set at *P* < 0.05.

## 3. Results

**Figure 1:**
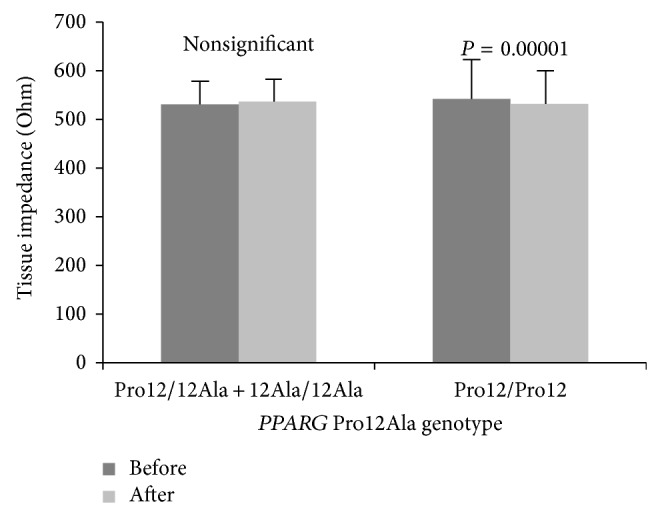
Changes in tissue impedance observed in participants (*PPARG* 12Ala carriers versus Pro12 homozygotes) before and after the completion of 12-week training program.

**Figure 2:**
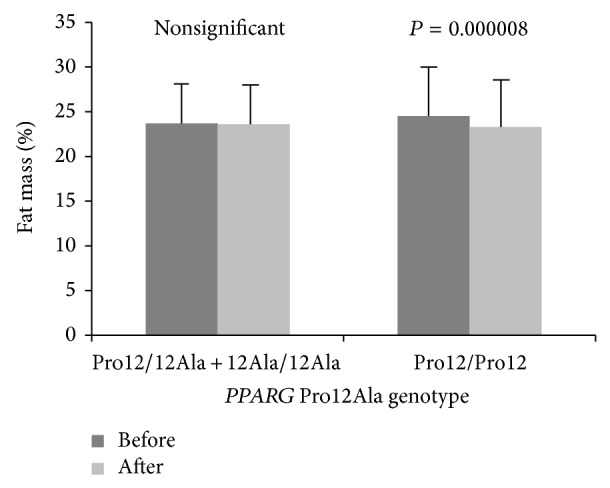
Changes in fat mass percentage observed in participants (*PPARG* 12Ala carriers versus Pro12 homozygotes) before and after the completion of 12-week training program.

**Figure 3:**
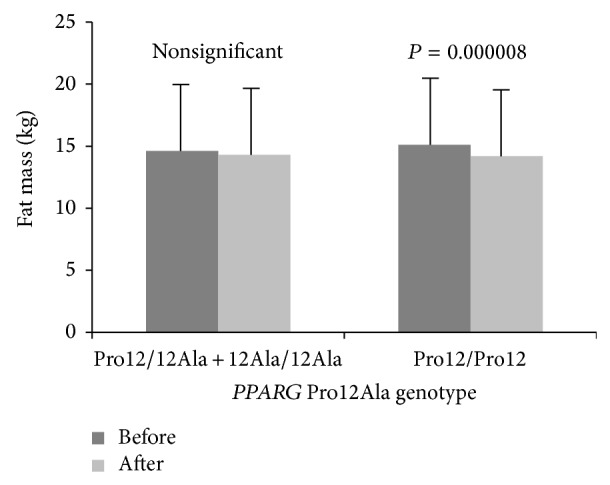
Changes in fat mass observed in participants (*PPARG* 12Ala carriers versus Pro12 homozygotes) before and after the completion of 12-week training program.

**Figure 4:**
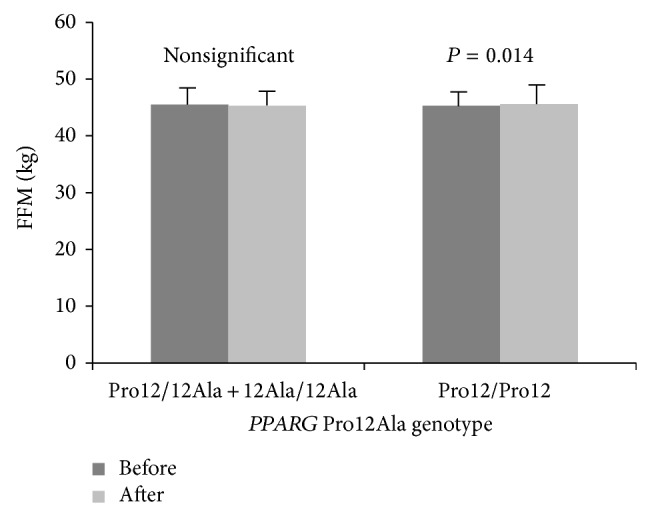
Changes in free fat mass (FFM) observed in participants (*PPARG* 12Ala carriers versus Pro12 homozygotes) before and after the completion of 12-week training program.

*PPARG* genotypes conformed to Hardy-Weinberg equilibrium (*P* = 0.549). To examine a hypothesis that the* PPARG* polymorphism modulates training response, we conducted a mixed 2 × 2 ANOVA with one between-subject factor (*PPARG* genotype: Pro12/Pro12 versus Pro12/12Ala + 12Ala/12Ala) and one within-subject factor (time: before training versus after training) for eight dependent variables (Table 1). There were five significant main effects of training: for body mass, *F*_(1,199)_ = 30.7, *P* < 0.0001, for BMI, *F*_(1,199)_ = 26.3, *P* = 0.000001, for BMR, *F*_(1,199)_ = 29.9, *P* < 0.0001, for fat mass percentage, *F*_(1,199)_ = 32.5, *P* < 0.0001, and for fat mass, *F*_(1,199)_ = 44.5, *P* < 0.0001. There was no main effect of* PPARG* genotypes on dependent variables, although five significant genotype × training interactions (for tissue impedance, fat mass percentage, fat mass, FFM, and TBW) were observed (Table 1), and after post hoc Tukey test four of them (for tissue impedance, fat mass percentage, fat mass, and FFM) remained statistically significant (Figures 1, 2, 3, and 4).

## 4. Discussion

Our study presents the results of genetic association study that aimed to test a hypothesis that the* PPARG* polymorphism modulates training response. To detect a correlation between the* PPARG* Pro12Ala genetic polymorphism and selected body composition measurements in participants undergoing training program, we examined the genotype distribution of the* PPARG* Pro12Ala allele in a group of 201 Polish women measured for selected body mass and body composition variables before and after the completion of a 12-week training period. We identified five significant main effects of performed training for different body mass measurements (body mass, BMI, BMR, fat mass, and fat mass percentage). However, with reference to interactions between genotype and training only four significant effects of* PPARG* genotypes on dependent variables were observed (for tissue impedance, fat mass percentage, fat mass, and FFM). The Pro12/Pro12 homozygotes displayed a greater fat mass and fat mass percentage as well as tissue impedance decrease with accompanying increase of free fat mass compared to 12Ala allele carriers.

These results indicate that the* PPARG* 12Ala variant may impair the training-induced positive effects on body mass measurements. Such hypothesis may be supported by the results of many previous studies showing the role of 12Ala allele in maintaining proper body mass. The body mass is undoubtedly determined by many genes, and studies concerning the assessment of the influence of individual genetic factors on the general BMI are very complex. A network of interactions between various genes affecting a studied feature in humans as well as many possible epigenetic and environmental factors (diet, applied training, lifestyle, medications taken, etc.) can modify the function of genetic factors. Considering that PPAR*γ* is a crucial element of the metabolic system that controls the body fat storage, it may be assumed that* PPARG* Pro12Ala polymorphism is one of such genetic factors. In consequence, its relevance in the context of susceptibility to obesity was of major interest. Numerous studies have attempted to find an association between the Pro12Ala polymorphism and body mass measurements/obesity; however, obtained results were inconsistent. Studies in healthy nonobese subjects revealed that* PPARG* gene locus is related to body mass index and lipid values (such as HDL and LDL) [23]. Regarding* PPARG* Pro12Ala polymorphism, there are publications reporting on positive association between higher BMI and 12Ala allele [24–28]; on the other hand, some authors indicated a lower BMI in 12Ala carriers [7, 29, 30]. Some studies suggested a higher risk of developing obese phenotype for 12Ala allele carriers, in both men [31] and women [32], but these results could not be replicated in further studies. One main finding of all these studies, including a meta-analysis of 40 datasets from 30 independent studies [33], was that the effects of carrying the* PPARG* 12Ala allele differ between overweight/obese and lean subjects [11, 24, 29]. The presence of* PPARG* 12Ala allele correlates with higher BMI only in individuals with marked obesity, while this effect was not observed in lean subjects [11].

It may be suggested that the Pro12Ala polymorphism exerts differential effects on BMI, probably due to the modifying impact of other genetic components and/or environmental factors, especially diet and training. When the diet is rich in polyunsaturated fatty acids the 12Ala allele favours BMI increases, but when the ratio of polyunsaturated to saturated fatty acids increases the reverse effect on BMI is observed [34]. Moreover, the effect of dietary fatty acid intake on BMI may be modified by physical activity. Interventional studies have revealed that associations of diet and activity level on fasting insulin differ between* PPARG* Pro12Ala genotypes. The beneficial additive effects of physical exercise and a healthy (i.e., rich in polyunsaturated fatty acids) diet were noticed only in homozygotes for the Pro12 allele. On the other hand, in individuals who were 12Ala allele carriers the relationships between healthy diet and appropriate physical training were more complicated and they did not correlate in the same additive manner. Magnitude of change in fasting insulin level observed in 12Ala allele carriers was only attenuated when both exposures of diet and activity were simultaneously elevated. When either polyunsaturated or saturated fatty acids ratio was elevated and, at the same time, the activity level was suppressed (and inversely) the fasting insulin levels in 12Ala carriers were similar to the highest insulin levels observed in Pro12/Pro12 individuals who were completely inactive and consumed a diet rich in saturated fatty acids [35]. Taking these findings into consideration,* PPARG* 12Ala allele might be included in the group of factors that are positively associated with a susceptibility to obesity; however, its presence in an individual's genotype is not sufficient to develop obesity, because the obese phenotype strongly depends on individual's lifestyle behaviours.

PPAR*γ* is known as a molecular sensor that is engaged in energy substrate selection by regulation of the metabolism and transport of fatty acids as well as glucose utilisation. Almost all of these metabolic effects are, at least partly, modulated and controlled by insulin-dependent signalling pathways. As it was mentioned earlier, the presence of the* PPARG* 12Ala allele in one's genotype sensitizes the tissue to the insulin action [7]. In consequence, 12Ala allele can influence lipid metabolism causing suppression of lipolysis in adipocytes and diminished release of free fatty acids [12] with accompanying increase of glucose uptake in human skeletal muscles [13, 14]. The observed correlation between carrying the 12Ala allele and diversity of metabolic pathways may be explained on the molecular level by the influence of Pro12Ala polymorphism on PPAR*γ* transcriptional activity.* In vitro* studies revealed that 12Ala PPAR*γ*2 variant ability to activate the PPRE sequences in artificial transfection constructs [36] as well as in promoters of target genes [7] was decreased, indicating that the* PPARG* 12Ala allele is associated with a less active form of PPAR*γ*2 protein. These results were confirmed* in vivo* in association studies. Changes in the expression of PPAR*γ* target genes depending on the Pro12Ala genotypes are diverse, reflecting different molecular effects of PPAR*γ* action. The* ADIPOQ* gene for adiponectin is normally positively regulated by PPAR*γ* and studying obese subjects revealed that plasma adiponectin levels were indeed significantly lower in 12Ala allele carriers [8]. Similar results were obtained in diabetic and coronary artery disease (CAD) patients carrying the* PPARG* 12Ala allele; plasma LPL activity was decreased in these patients [9]. Conversely, for genes that are negatively regulated by PPAR*γ* (e.g.,* LEP* gene for leptin), the opposite effect was observed in diabetic patients, with higher plasma leptin levels in 12Ala allele subjects [10].

These alterations in the activity of the PPAR*γ*2 12Ala variant and associated different physiological effects may result from the specific localisation of the Pro12Ala amino acid change in the PPAR*γ* molecule. This amino acid substitution was positioned within the AF-1 domain region that was proved to be involved in the control of PPAR*γ* transcriptional activity (independent of ligand binding) via consensus MAPK (mitogen-activated protein kinase) site [37]. The chemical modifications (such as phosphorylation or sumoylation) of MAPK site result in reduction of PPAR*γ*2 ligand-binding affinity by recruiting a repressor protein to the modified AF-1 region [38] or by intramolecular communication between AF-1 and the ligand biding domain (LBD) [39]. Consequently, the potential of liganded PPAR*γ*2 to activate the transcription of its target genes is decreased. The detailed association between the Pro12Ala polymorphism and the described above negative modulation of the PPAR*γ* activity remains unclear, because position 12 in the PPAR*γ* protein is not a consensus site for phosphorylation or sumoylation. The possible explanation may lie in intramolecular interactions between different amino acids of the AF-1 domain that indirectly facilitate the phosphorylation and/or sumoylation processes [19]; however, it still needs to be confirmed in more detailed analyses. It is worth noticing that some authors suggested there may be other functional polymorphisms in* PPARG* gene regulatory regions (presumably in linkage disequilibrium with the Pro12Ala polymorphic site) that could decrease PPAR*γ* transcriptional activity [40].

## 5. Conclusions

All the data presented above shed some light on the modifying role played by* PPARG* Pro12Ala polymorphism in differentiating the beneficial effects of physical activity between the specific Pro12Ala genotype carriers. The results of our experiment suggest that* PPARG* genotype can modulate training-induced body mass measurements changes. We have displayed that after completion of the training program the Pro12/Pro12 homozygotes were characterised by a greater decrease of body fat mass measurements in comparison with 12Ala allele carriers. From this evidence, it could be concluded that the 12Ala variant may be considered a disadvantageous factor in the context of training-induced positive effects on body mass measurements.

We are aware that our study has some limitations. All of the results received during genetic associations studies should be interpreted with caution, especially considering diverse data obtained for the correlations of obesity predisposition and* PPARG* Pro12Ala polymorphism in different human populations. This problem has been brought up in many studies and in most cases the conclusion is that the variation within the* PPARG* gene does not influence any physiological traits alone. Even if the Pro12Ala polymorphism actually modulates body weight, probably only small part of the total variance of BMI can be explained by this polymorphism, because of the polygenic character of this trait, implying that multiple gene-environment interactions may contribute to the observed differential effects.

## Figures and Tables

**Table 1:** *PPARG* genotypes and response to training (analyzed by two-way mixed ANOVA test).

Parameter	12Ala/12Ala + Pro12/12Ala (*n* = 60)		Pro12/Pro12 (*n* = 141)		Genotype	Training	Genotype × training
	Before training	After training	Before training	After training			
Body mass (kg)	60.1 ± 6.8	59.6 ± 6.9	60.3 ± 6.3	59.8 ± 6.4	*P* = 0.819	*P* < 0.0001	*P* = 0.481
BMI (kg × m^−2^)	21.5 ± 2.5	21.3 ± 2.5	21.8 ± 2.5	21.6 ± 2.4	*P* = 0.500	*P* = 0.000001	*P* = 0.371
BMR (kJ)	6041 ± 301	6024 ± 304	6044 ± 262	6021 ± 267	*P* = 0.992	*P* < 0.0001	*P* = 0.418
Tissue impedance (Ohm)	550 ± 47	556 ± 50	556 ± 65	544 ± 61	*P* = 0.749	*P* = 0.166	*P* = 0.0001
Fat mass percentage (% FM)	23.7 ± 5.0	23.6 ± 4.7	24.5 ± 5.0	23.3 ± 5.3	*P* = 0.763	*P* < 0.0001	*P* = 0.00003
Fat mass (kg)	14.6 ± 4.8	14.3 ± 4.7	15.1 ± 4.3	14.2 ± 4.6	*P* = 0.782	*P* < 0.0001	*P* = 0.0002
FFM (kg)	45.5 ± 2.5	45.3 ± 2.6	45.3 ± 2.5	45.6 ± 2.5	*P* = 0.908	*P* = 0.678	*P* = 0.005
TBW (kg)	33.3 ± 1.8	33.1 ± 1.9	33.2 ± 1.9	33.4 ± 1.8	*P* = 0.806	*P* = 0.902	*P* = 0.005

Mean ± standard deviation; *P* values for main effects (genotype and training) and genotype × training interactions.
